# Genetic polymorphisms in *FABP2*, *CYP2E1*, and *TP53* genes are potentially associated with colorectal cancer susceptibility

**DOI:** 10.1038/s41598-024-70381-y

**Published:** 2024-09-03

**Authors:** Maryam Ijaz, Chien-Chin Chen, Rana Khalid Iqbal, Hafiza Aneela Farooq, Rubaida Mehmood, Muhammad Asif, Atif Akbar, Adil Khan, Waseem Ijaz, Mourad Ben Said, Gezahign Fentahun Wondmie, Samir Ibenmoussa, Mohammad K. Okla, Furhan Iqbal

**Affiliations:** 1 Institute of Zoology, Bahauddin Zakariya University, Multan, 60800 Pakistan; 2 Department of Pathology, Ditmanson Medical Foundation Chia-Yi Christian Hospital, Chiayi, 60002 Taiwan; 3 Department of Cosmetic Science, Chia Nan University of Pharmacy and Science, Tainan, 71710 Taiwan; 4 Department of Biotechnology and Bioindustry Sciences, National Cheng Kung University, Tainan, 701 Taiwan; 5 Ph.D. Program in Translational Medicine, Rong Hsing Research Center for Translational Medicine, National Chung Hsing University, Taichung, 40227 Taiwan; 6 Institute of Molecular Biology and Biotechnology, Bahauddin Zakariya University, Multan, 60800 Pakistan; 7 Department of Biology, University of Padova, Padua, Italy; 8 Minar Multan Institute of Nuclear Medicine and Radiology Hospital, Multan, 60800 Pakistan; 9 Department of Statistics, Bahauddin Zakariya University, Multan, 60800 Pakistan; 10 Department of Botany and Zoology, Bacha Khan University, Charsadda, 24420 Khyber Pakhtunkhwa Pakistan; 11 University of Veterinary and Animal Sciences, Lahore, 54000 Pakistan; 12 Laboratory of Microbiology, National School of Veterinary Medicine of Sidi Thabet, University of Manouba, 2010 Manouba, Tunisia; 13 Department of Basic Sciences, Higher Institute of Biotechnology of Sidi Thabet, University of Manouba, 2010 Manouba, Tunisia; 14 Department of Biology, Bahir Dar University, P.O.Box 79, Bahir Dar, Ethiopia; 15 Laboratory of Therapeutic and Organic Chemistry, Faculty of Pharmacy, University of Montpellier, 34000 Montpellier, France; 16 Botany and Microbiology Department, College of Science, King Saud University, P.O. Box 2455, 11451 Riyadh, Saudi Arabia

**Keywords:** Colorectal cancer, Genetic polymorphisms, FABP2 gene, CYP2E1 gene, TP53 gene, Pakistani population, Biochemistry, Cancer, Genetics

## Abstract

Colorectal cancer (CRC) is among the most prevalent cancers with a high mortality rate. Both genetic and environmental factors contribute to CRC development. This study aimed to assess the association of single nucleotide polymorphisms (SNPs) in the fatty acid binding protein-2 (rs1799883), Cytochrome P450 2E1 (rs3813865), TP53 (rs1042522), and Murine double minute 2 (rs1042522) genes with CRC. A cross-sectional case–control study was conducted at the Institute of Molecular Biology and Biotechnology from May 2020 to March 2021, involving CRC patients (N = 100) and controls (N = 100) recruited from the Multan district in Pakistan. Polymerase chain reaction-restriction fragment length polymorphism (PCR–RFLP) and tetra-primer amplification refractory mutation system-polymerase chain reaction (ARMS-PCR) were employed to investigate the studied SNPs. The association of SNPs in all genes with CRC was examined either individually or in various combinations. Genotypes at three SNPs, rs1799883 in FABP2, rs3813865 in CYP2E1, and rs1042522 in TP53, were found to be associated with the development of CRC, while rs1042522 in MDM2 was not. Patients who were married, smoked, lacked exercise habits or had a family history of CRC were at a greater risk of acquiring the disease. FABP2 gene rs1799883, CYP2E1 gene rs3813865, and TP53 gene rs1042522 polymorphisms are significant in the development of CRC in Pakistani participants.

## Introduction

Colorectal cancer (CRC), ranking as the third most frequently diagnosed cancer worldwide with a notable mortality rate^1^, is influenced by a combination of germline mutations in high penetrance genes (60% cases) and lifestyle-related factors (40% cases)^2,3^. The individual susceptibility to CRC is intricately tied to genetic polymorphisms, wherein certain variations exhibit noteworthy ethnic disparities, reflecting diverse frequencies of mutant genotypes across populations^4–6^.

Numerous studies have identified specific genes and their single nucleotide polymorphisms (SNPs) associated with CRC in various ethnic groups. For instance, the FABP2 gene, encoding the fatty acid-binding protein 2 (FABP2), demonstrates a crucial role in the uptake, transport, and regulation of fatty acids, with polymorphisms in this gene linked to insulin resistance a well-established CRC risk factor in Han Chinese populations^7^. This emphasizes the intricate interplay between genetic variations, lifestyle factors, and CRC susceptibility, shedding light on the diverse molecular landscape of this complex disease.

The delicate equilibrium between xenobiotic absorption and elimination plays a crucial role in safeguarding against deoxyribonucleic acid (DNA) damage induced by chemical carcinogens. A well-established fact is that both exogenous (xenobiotics) and endogenous chemical carcinogens necessitate biotransformation into activated forms to manifest their carcinogenic potential^8^.

The Cytochrome P450 2E1 (*CYP2E1*) gene encodes an enzyme within the cytochrome P450 superfamily, pivotal for the detoxification and activation of various low molecular compounds. These encompass ethanol, acetone, and pharmaceuticals like acetaminophen, isoniazid, chlorzoxazone, and fluorinated anesthetics, as well as several procarcinogens such as benzene, N-nitrosodimethylamine, and styrene^4^. Beyond detoxification, these enzymes frequently facilitate the metabolic activation of procarcinogens into their ultimate carcinogenic forms^9^. Mutations in the *CYP2E1* gene, responsible for encoding the CYP2E1 enzyme, can yield enzyme variants with varying activity levels-higher, lower, or absent. Consequently, tailoring therapy based on individual’s *CYP2E1* genotype has the potential to enhance drug efficacy^4^.

Reportedly, a myriad of mutations (approximately 200) and aberrant expression in the *TP53* gene have been implicated in various cancers^10^. Epidemiological evidence underscores that *TP53* mutations are prevalent in around half of all colorectal cancers^11,12^. The *TP53* gene encodes the p53 protein, a transcription factor pivotal for maintaining genomic integrity through surveillance of cell cycle progression and cell survival^13^. Renowned as the 'guardian of the genome,' the p53 protein is recognized for stimulating the expression of multiple pro-apoptotic signaling molecules, including FOXO1, FOXO3, and TRAIL, while concurrently orchestrating DNA repair and cell division regulation^14^.

Additionally, p53 is known to activate the murine double minute 2 (MDM2) protein at the transcriptional level. MDM2, in turn, acts as a crucial negative regulator of *TP53* by intensifying proteasomal degradation, particularly when there is an excess of p53 protein^15^. This intricate relationship suggests that MDM2 overexpression may impede p53 function, allowing damaged cells to evade cell cycle checkpoint control and potentially evolve into carcinogenic entities^16^.

While the association of single nucleotide polymorphisms (SNPs) in *TP53, MDM2, CYP2E1*, and *FABP2* with colorectal cancer (CRC) has been explored in various populations, there remains a critical void in the literature pertaining to such investigations in the Pakistani context. Initially classified as a low-risk zone for CRC, recent findings by Bhurgri et al.^17^ have documented a notable surge in CRC cases among individuals aged 50 and above in Pakistan. This increased risk is attributed to factors such as the elevated consumption of preserved foods, and animal products, smoking, excessive alcohol intake, and inflammatory bowel disease. Compounding this, diminished physical activity levels coupled with a significant rise in obesity prevalence further contribute to the heightened CRC risk within the Pakistani population^18^.

Despite recommendations advocating clinical and genotypic screening as an integral part of future health sector planning, particularly focusing on high-risk younger age groups, implementation of these suggestions has been lacking^17^. Recognizing this gap, the current study seeks to address whether the examined SNPs, including rs1799883 in *FABP2*, rs3813865 in *CYP2E1*, rs1042522 in *TP53*, and rs2279744 in *MDM2*, individually or in combination, are correlated with CRC in the Pakistani population. By doing so, we aim to provide crucial insights into the genetic determinants of CRC susceptibility specific to Pakistan, thereby contributing to the development of targeted preventive and therapeutic strategies for this population.

## Materials and methods

### Subjects and data collection

The cross-sectional case–control study was conducted at the Institute of Molecular Biology and Biotechnology from May 2020 to March 2021, encompassing clinically confirmed colorectal cancer (CRC) patients and healthy controls. Blood samples from CRC patients (N = 100) were procured from the Multan Institute of Nuclear Medicine and Radiotherapy (MINAR) and the Oncology ward of Nishter Medical University and Hospital, Multan, Pakistan. Patient diagnosis relied on carcinoembryonic antigen (CEA) levels and histological evidence obtained through endoscopic biopsy. Enrolled subjects, representing diverse cities in Southern Punjab, exhibited variations in ethnic origins, gender, and age. Controls (N = 100) were age-matched to cases and exhibited no colorectal cancer or systemic illnesses. Sample size estimation during random collection employed Solvin’s formula, calculated as follows:

$$ {\text{n }} = {\text{ N/}}\left( {{1} + {\text{N }}*{\text{ e}}^{{2}} } \right). $$

whereas: n = no. of samples, N = total population, and e = margin of error^19^.

After obtaining informed consent, a comprehensive questionnaire was administered to all participants to gather essential epidemiological data, covering aspects such as age, gender, marital status, family history, and habits related to smoking and exercise.

### Blood collection and DNA extraction

A blood sample of 3–5 ml was collected from each participant and stored at − 4 °C in Ethylene Diamine Tetra Acetic acid (EDTA)-coated vials until further analysis. DNA extraction from the whole blood was performed using an inorganic DNA extraction protocol, as detailed by Arshad et al.^19^.

### Amplification and genotyping of rs1799883 in *FABP2*

For genotyping the Ala54Thr SNP (rs1799883) in the *FABP2* gene, a polymerase chain reaction-restriction fragment length polymorphism (PCR–RFLP) approach was employed. Oligonucleotide primers, as per Baier et al.^20^ were utilized for amplification: forward primer 5ʹ-ACAGGTGTTAATATAGTGAAAAG-3ʹ and reverse primer 5ʹ-TACCCTGAGTTCAGTTCCGTC-3. A reaction mixture of 20 µl was prepared that contained 13 mM Tris–HCl (pH 8.3), 65 mM KCl, 1.5 mM MgCl_2_, 300 µM of each dNTP,1U of DNA Polymerase (Thermo Scientific, United States), 0.5 µM of each primer and 5 µl of template DNA^20^. The amplification of DNA was performed using a thermal cycler (Gene Amp™ PCR system 2700, Applied Biosystems Inc., United Kingdom). The thermo-profile included an initial denaturation at 95 °C for 3 min, followed by 30 cycles of denaturation at 95 °C for 30 s, annealing at 55 °C for 30 s, and elongation at 72 °C for 45 s. The final extension was carried out for 3 min at 72 °C^21^. Subsequently, the amplified product underwent restriction using *Hha1*, a restriction enzyme isolated from *Haemophilus haemolyticus* bacteria (New England BioLabs, USA). This step was conducted in a 20 µl reaction mixture composed of 10 µl PCR product, 2 µl CutSmart buffer, and 7 µl double-distilled water, maintained at 37 °C for 1 h^21^. The resolution of the restriction products was achieved using a 2% agarose gel and visualized on an ultraviolet (UV) transilluminator (Biostep, Germany).

### Amplification and genotyping of rs3813865 in *CYP2E1*

A T-ARMS–PCR was employed to amplify rs3813865 (G/C), a single nucleotide polymorphism (SNP) in the *CYP2E1* gene, following the protocol outlined by Suhda et al.^9^. The primers utilized in this T-ARMS PCR were as follows: outer forward 5ʹ TGA TGT TGG TTG GGC ATC TA 3ʹ, outer reverse 5′ CCTCGA GGT GAG AAC TGA CA 3′, inner forward 5ʹ CTC ACC CCA CCA AAG CCT AC 3ʹ, inner reverse 5ʹ-CCA CAG ACT GAA ATT GAA CCC 3ʹ. A reaction mixture of 25 µl was prepared that contained 13 mM Tris–HCl (pH 8.3), 65 mM KCl, 2 mM MgCl_2_, 300 µM of each dNTP,1U of DNA Polymerase (Thermo Scientific, United States), 0.5 µM of each primer and 5 µl of template Subsequently, the amplification of rs3813865 in CYP2E1 was conducted under the following thermal conditions: initial denaturation for 5 min at 95 °C, followed by 30 cycles of denaturation for 30 s at 95 °C, annealing for 30 s at 56 °C, elongation for 30 s at 72 °C, and a final extension for 7 min at 72 °C^9^.

### Amplification and genotyping of rs1042522 in *TP53*

A T-ARMS–PCR analysis was performed to investigate the rs1042522 (G/C) single nucleotide polymorphism (SNP) in the *TP53* gene, utilizing the methodology outlined by Asadi et al.^11^. The primers employed in this T-ARMS PCR were as follows: outer forward 5ʹ TGCAGGGGGATACGGCCAGGCATTGAAGTC 3ʹ, outer reverse 5ʹ TGGGGGGCTGAGGACCTGGTCCTCT 3ʹ, inner forward 5ʹ GCTGCTGGTGCAGGGGCCAGGG 3ʹ, inner reverse 5ʹ CCAGAATGCCAGAGGCTGCTCCGCG 3ʹ. A reaction mixture of 25 µl was prepared that contained 13 mM Tris–HCl (pH 8.3), 65 mM KCl, 1.5 mM MgCl_2_, 300 µM of each dNTP,1U of DNA Polymerase (Thermo Scientific, United States), 0.5 µM of each primer and 5 µl of template DNA. For the amplification of rs1042522, the thermal profile included an initial denaturation for 10 min at 95 °C, followed by 25 cycles of denaturation at 95 °C for 45 s, annealing at 56 °C for 30 s, elongation at 72 °C for 45 s, and a final extension for 10 min at 72 °C^11^.

### Amplification and genotyping of rs2279744 in *MDM2*

A T-ARMS–PCR analysis was carried out to investigate the rs2279744 (G/T) single nucleotide polymorphism (SNP) in the *MDM2* gene, following the protocol outlined by Xiao et al.^22^. The primers utilized in this T-ARMS PCR were as follows: outer forward 5ʹ GGC AGTCGCCGCCAGGGAGGAGGGCGG 3ʹ, outer reverse 5ʹ TGCCCACTGAACCGGCCCAATCCCGCCCAG 3ʹ, inner forward 5ʹ GGGGGCCGGGGGCTGCGGGGCCGTTT 3ʹ, inner reverse 5ʹ ACCTGCGATCATCCGGACCTCCCGCGCTGC 3ʹ. A reaction mixture of 25 µl was prepared that contained 13 mM Tris–HCl (pH 8.3), 65 mM KCl, 1.5 mM MgCl_2_, 300 µM of each dNTP,1U of DNA Polymerase (Thermo Scientific, United States), 0.5 µM of each primer and 5 µl of template For the amplification of rs2279744, the thermal profile comprised an initial denaturation for 10 min at 95 °C, followed by 25 cycles of denaturation at 95 °C for 45 s, annealing at 52 °C for 30 s, elongation at 72 °C for 45 s, and a final extension for 10 min at 72 °C^22^.

### Statistical analysis

The analysis of data was performed using Minitab version 18 (Minitab, USA). Genotypic frequencies were ascertained through direct counting. The chi-square test was employed to compare genotype and allelic frequencies between cases and controls, as well as to assess the correlation between colorectal cancer (CRC) and the investigated risk factors. All statistical tests were two-tailed, with a significance level set at P < 0.05 to establish statistical significance. Odds ratio and adjusted odds ratio were calculated where required.

### Ethics approval and consent to participate

Ethical Research Committee of the Institute of Pure and Applied Biology at Bahauddin Zakariya University Multan (Pakistan) approved all the experimental procedures and protocols applied in this study via letter number IP&AB/Ethics/41/2020. All the subject handling procedures and experimental protocols were performed in accordance with the Declaration of Helsinki. Informed consent was obtained from all subjects involved in the study.

## Results

### Genotypic and allelic frequency at rs1799883 in *FABP2* and their association with CRC

The PCR successfully amplified a 180 bp amplicon from *FABP2*. Subsequent restriction with *Hha1* resulted in a distinct pattern: homozygous dominant individuals (GG) exhibited two bands (81 bp and 99 bp), heterozygous (GA) subjects displayed three bands (81 bp, 99 bp, and 180 bp), and the uncut 180 bp product indicated homozygous mutant individuals (AA) (Supplementary Fig. 1A). Data analysis demonstrated significant variation in both genotypes and allelic frequencies at rs1799883 in FABP2 when comparing the case and control groups (P = 0.05). Notably, cases exhibited a higher frequency of the homozygous dominant (GG) genotype, whereas the control group had a higher frequency of the heterozygous (GA) genotype. The odd ratio (OR) of 1.47 is indicating that cases have 1.47 fold higher risk than controls to develop CRC (Table 1).

**Table 1:** Distribution of genotypic and allelic frequencies of rs1799883 in *FABP2*, rs3813865 in *CYP2E1*, rs1042522 in *TP53,* and rs2279744 in *MDM2* gene among cases and controls and their possible association with colorectal cancer.

Gene and SNP	Case/control	Genotypic frequency			Allelic frequency		Chi-square value	P-value	OR (C.I.)	AO (C.I.)R
*FABP2* rs1799883		GG	GA	AA	G	A				
	Control	29 (29%)	46 (46%)	25 (25%)	0.52	0.48	5.305	0.05*	1.0 (Ref.)	1.0 (Ref.)
	Case	44 (44%)	33 (33%)	23 (23%)	0.6	0.4			1.47 (0.98–2.22)	1.53 (1.01–2.32)
*CYP2E1* rs3813865		GG	GC	CC	G	C				
	Control	27 (27%)	17 (17%)	56 (56%)	0.36	0.64	10.131	0.006**	1.0 (Ref.)	1.0 (Ref.)
	Case	28 (28%)	35 (35%)	37 (37%)	0.46	0.54			1.56 (1.02–2.37)	1.64 (1.07–2.52)
*TP53* rs1042522		GG	GC	CC	G	C				
	Control	45 (45%)	50 (50%)	5 (5%)	0.68	0.32	14.655	0.05*	1.0 (Ref.)	1.0 (Ref.)
	Case	63 (63%)	26 (26%)	11 (11%)	0.26	0.74			3.50 (2.14–5.73)	3.75 (2.27–6.18)
*MDM2* rs2279744		TT	TG	GG	T	G				
	Control	30 (30%)	9 (9%)	61 (61%)	0.35	0.65	0.225	1	1.0 (Ref.)	1.0 (Ref.)
	Case	29 (29%)	11 (11%)	60 (60%)	0.35	0.65			0.97 (0.65–1.45)	0.95 (0.63–1.43)

Percentage frequency is given in parentheses. The P-value represents the outcome of the Chi-square test calculated for each single nucleotide polymorphism (SNPs). OR is odds ratio and AOR is adjusted odds ratio calculated for each SNP. *P < 0.05; **P < 0.01. *C.I.* 95% confidence interval.

### Genotypic and allelic frequency at rs3813865 in *CYP2E1* and their association with CRC

The Tetra-primer Amplification Refractory Mutation System–Polymerase Chain Reaction (T-ARMS–PCR) generated a 499 bp amplicon for the outer primers, 303 bp for homozygous wild type (GG), and 236 bp for homozygous mutant (CC), while the heterozygous (GC) configuration produced all three bands of 499, 303, and 236 bp (Supplementary Fig. 1B). Analysis of genotypes and allelic frequency distribution unveiled significant variation (P = 0.006) in genotypic frequencies at rs3813865 in *CYP2E1* when comparing patients and controls. Notably, the frequency of heterozygous alleles (GC) was higher in CRC patients, whereas homozygous mutant alleles (CC) were more prevalent in the control group. The OR values are indicating that cases has 1.56 fold more risk than controls to develop CRC (Table 1).

### Genotypic and allelic frequency at rs1042522 in* TP53* and their association with CRC

The T-ARMS–PCR yielded a 493 bp amplicon for the outer primers, 247 bp for homozygous wild type (CC), and 200 bp for homozygous mutant (GG), while the heterozygous (GC) configuration produced all three bands of 493, 247, and 200 bp (Supplementary Fig. 1C). Genotypic and allelic frequency analysis uncovered significant variation (P = 0.05) in genotypic frequencies at rs1042522 in TP53 when comparing patients and controls. Remarkably, the frequency of homozygous mutant alleles (GG) was higher in CRC patients, whereas heterozygous alleles (GC) were more prevalent in the control group. OR analysis revealed that cases had 3.5 fold higher risk to develop CRC than controls (Table 1).

### Genotypic and allelic frequency at rs2279744 in *MDM2* and their association with CRC

The T-ARMS–PCR produced a 224 bp amplicon for the outer primers, 158 bp for homozygous wild type (GG), and 122 bp for homozygous mutant (TT), while the heterozygous (GT) configuration generated all three bands of 224, 158, and 122 bp (Supplementary Fig. 1D). Analysis of genotypic distribution and allelic frequencies indicated that none of the genotypes at rs2279744 in *MDM2* were found to be associated with CRC (P = 1). OR analysis indicated that both case and controls had equal susceptibility to develop CRC (OR = 0.97) (Table 1).

### Interactions between SNPs and their association with CRC

Analysis of genotype frequencies revealed that certain combinations of genotypes at two or more single nucleotide polymorphisms (SNPs) significantly increased the risk of developing colorectal cancer (CRC). Protective combinations, with significantly higher frequencies among controls, contrasted with higher-risk combinations more prevalent among cases (Table 2). Comparing the genotype combination of rs1799883 (*FABP2*) and rs3813865 (*CYP2E1*) revealed that individuals with the wild type (GG) at *FABP2* and the heterozygous (GC) genotype at *CYP2E1* had a significantly higher incidence of CRC development (P = 0.02) (Table 2). Similarly, comparing rs1042522 (*TP53*) and rs2279744 (*MDM2*) genotype combinations demonstrated that individuals with the wild type (GG) at *TP53* and the mutant (TT) genotype at MDM2 had a significantly higher incidence of CRC development (P = 0.05) (Table 2). The most CRC-susceptible genotypes were observed when different genotype combinations of rs3813865 (*CYP2E1*) and rs1042522 (*TP53*) were examined. Specifically, all three genotypes of CYP2E1 at rs3813865 were found to be susceptible to CRC when present with either wild-type (GG) or heterozygous (GC) genotypes at rs1042522 in TP53 (P = 0.04) (Table 2). However, when all genotypes of the four studied SNPs were analyzed in combination, no specific combination types were found to be susceptible to developing CRC (P = 0.801) (Table 2).

**Table 2:** Genotype frequency distribution among cases and controls for various combinations at *FABP2* rs1799883, *CYP2E1* rs3813865, *TP53* rs1042522, and *MDM2* rs2279744 and their possible association with colorectal cancer.

Genotype	Case	Controls	Chi-square value	*P*-value
*FABP2* rs1799883 and *CYP2E1* rs3813865				
* FABP2* (GG) and *CYP2E1* (GG)	9	6	17.873	0.02*
* FABP2* (GG) *CYP2E1* (GC)	17	4		
* FABP2* (GG) *CYP2E1* (CC)	15	19		
* FABP2* (GA) and *CYP2E1* (GG)	9	13		
* FABP2* (GA) *CYP2E1* (GC)	13	9		
* FABP2* (GA) *CYP2E1* (CC)	9	24		
* FABP2* (AA) and *CYP2E1* (GG)	10	8		
* FABP2* (AA) *CYP2E1* (GC)	5	4		
* FABP2* (AA) *CYP2E1* (CC)	11	13		
*TP53* rs1042522 and *MDM2* rs2279744				
* TP53* (GG) and *MDM2* (TT)	20	11	15.265	0.05*
* TP53* (GG) and *MDM2* (TG)	7	5		
* TP53* (GG) and *MDM2* (GG)	36	29		
* TP53* (GC) and *MDM2* (TT)	7	16		
* TP53* (GC) and *MDM2* (TG)	2	4		
* TP53* (GC) and *MDM2* (GG)	17	31		
* TP53* (CC) and *MDM2* (TT)	2	3		
* TP53* (CC) and *MDM2* (TG)	2	1		
* TP53* (CC) and *MDM2* (GG)	7	2		
*FABP2* rs1799883 and *MDM2* rs2279744				
* FABP2* (GG) and *MDM2* (TT)	12	11	7.228	0.512
* FABP2* (GG) *MDM2* (TG)	3	2		
* FABP2* (GG) *MDM2* (GG)	26	16		
* FABP2* (GA) and *MDM2* (TT)	11	12		
* FABP2* (GA) *MDM2* (TG)	4	3		
* FABP2* (GA) *MDM2* (GG)	17	31		
* FABP2* (AA) and *MDM2* (TT)	6	7		
* FABP2* (AA) *MDM2* (TG)	3	4		
* FABP2* (AA) *MDM2* (GG)	16	14		
*CYP2E1* rs3813865 and *TP53* rs1042522				
* CYP2E1* (GG) and *TP53* (GG)	18	10	8.362	0.04*
* CYP2E1* (GG) and *TP53* (GC)	9	1		
* CYP2E1* (GG) and *TP53* (CC)	2	1		
* CYP2E1* (GC) and *TP53* (GG)	28	8		
* CYP2E1* (GC) and *TP53* (GC)	6	8		
* CYP2E1* (GC) and *TP53* (CC)	1	1		
* CYP2E1* (CC) and *TP53* (GG)	17	27		
* CYP2E1* (CC) and *TP53* (GC)	12	1		
* CYP2E1* (CC) and *TP53* (CC)	8	1		
*FABP2* rs1799883, *CYP2E1* rs3813865, *TP53* rs1042522, and *MDM2* rs2279744				
* FABP2* (GG), *CYP2E1* (GG), *TP53* (GG) and *MDM2* (TT)	2	1	0.064	0.801
* FABP2* (GG), *CYP2E1* (GC), *TP53* (GC) and *MDM2* (TG)	0	0		
* FABP2* (GG), *CYP2E1* (CC), *TP53* (CC) and *MDM2* (GG)	4	1		
* FABP2* (GA), *CYP2E1* (GG), *TP53* (GG) and *MDM2* (TT)	3	2		
* FABP2* (GA), *CYP2E1* (GC), *TP53* (GC) and *MDM2* (TG)	0	0		
* FABP2* (GA), *CYP2E1* (CC), *TP53* (CC) and *MDM2* (GG)	0	0		
* FABP2* (AA), *CYP2E1* (GG), *TP53* (GG) and *MDM2* (TT)	1	1		
* FABP2* (AA), *CYP2E1* (GC), *TP53* (GC) and *MDM2* (TG)	0	0		
* FABP2* (AA), *CYP2E1* (CC), *TP53* (CC) and *MDM2* (GG)	1	1		

*P < 0.05; **P < 0.01.

### Association of Risk Factors and CRC

Upon analyzing various clinical risk factors, significant differences were observed in marital status (P < 0.001), smoking (P < 0.001), family history (P = 0.002) and exercise habits (P = 0.01) when comparing controls to patients with CRC (Table 3). Additionally, there were no statistically significant differences in age and gender (P > 0.05). Odd ratio analysis indicated that married subjected (OR = 1), smokers (OR = 5.27), subjects having family history (OR = 2.64) and those do not have exercise habit (OR = 1) had higher susceptibility to develop CRC during present investigation (Table 3).

**Table 3:** Analysis of the clinical risk factors associated with colorectal cancer.

Risk factor	Categories	Case (N = 100)	Control (N = 100)	Odds ratio (C.I.)	*P*-value
Age (years)	< 50	59 (59%)	59 (59%)	1.0 (Ref.)	0.9
	≥ 50	41 (41%)	41 (41%)	1.05 (0.61–1.81)	
Gender	Male	70 (70%)	58 (58%)	1.641 (0.93–2.88)	0.09
	Female	30 (30%)	42 (42%)	1.0 (Ref.)	
Marital status	Single	59 (59%)	90 (90%)	0.17 (0.08–0.34)	P < 0.001***
	Married	41 (41%)	10 (10%)	1.0 (Ref.)	
Smoking	Yes	57 (57%)	12 (12%)	5.27 (2.65–10.49)	P < 0.001***
	No	43 (43%)	88 (88%)	1.0 (Ref.)	
Family history	Yes	41 (41%)	20 (20%)	2.64 (1.43–4.88)	0.002**
	No	59 (59%)	80 (80%)	(Ref.)	
Exercise habit	Yes	56 (56%)	72 (72%)	0.48 (0.27–0.84)	0.01**
	No	44 (44%)	28 (28%)	1.0 (Ref.)	

N represents the total number of samples in each category, and percentages are given in parentheses. P value indicates the chi-square test results when each parameter was compared between the control and patients. *P < 0.05; **P < 0.01; ***P < 0.001. *C.I.*: 95% confidence interval.

## Discussion

The incidence of colorectal cancer (CRC) is on the rise in Pakistan, yet the country lacks a specific CRC control program or a national cancer registry. Due to limited health care facilities, especially in rural areas, diagnosis of CRC patients in Pakistan is often delayed and usually patients are diagnosed at an advanced stage of the disease^23^. Moreover, there is a dearth of data in the literature regarding the genetic determinants of CRC in the Pakistani population. A comprehensive literature review identified only one study reporting an association between the deletion of the Glutathione S transferase M1 gene and the risk of CRC development in individuals from Khyber Pakhtunkhwa, Pakistan^24^. Given this gap in knowledge, the present study was undertaken to investigate the association of genotypes at rs1799883 in *FABP2*, rs3813865 in *CYP2E1*, rs1042522 in *TP53*, and rs2279744 in *MDM2*, both individually and in various combinations, with the development of CRC in subjects enrolled from Punjab, Pakistan.

The FABP2 protein plays a crucial role in the absorption and intracellular transport of long-chain fatty acids. The rs1799883 single nucleotide polymorphism (SNP) in the *FABP2* gene leads to a threonine-for-alanine substitution, altering the structure and function of FABP2^25^. Individuals with this genetic variant exhibit increased fatty acid uptake in the intestinal lumen, as the variant protein binds to fatty acids with double the affinity compared to the wild-type protein^26^. Our analysis revealed an association between rs1799883 in the *FABP2* gene and colorectal cancer (CRC), with cases exhibiting a higher frequency of the homozygous dominant (GG) genotype, while controls had a higher frequency of the heterozygous (GA) genotype (Table 1).

In contrast to our findings, studies by Andersen et al.^27^, Hu et al.^7^, and Kato et al.^28^ reported no association between any genotypes at rs1799883 in *FABP2* and CRC development in Danish, Han Chinese populations, and residents of the Metropolitan Detroit Tri-County area in the US. This observed discrepancy in results emphasizes the potential role of ethnicity in CRC. Additionally, the genotyping technique employed for the studied SNP may contribute to the variation in results.

Susceptibility to colorectal cancer (CRC) varies among individuals due to differences in their metabolism and detoxification potential of gastrointestinal carcinogens. Environmental and genetic factors both play significant roles in CRC development^24^. Our results demonstrated a significantly higher frequency of heterozygous alleles (GC) in CRC patients compared to controls at rs3813865 in *CYP2E1*, indicating an association with CRC incidence (Table 1). However, the association between rs3813865 in *CYP2E1* and CRC has been observed in a limited number of studies. Our findings align with Tang et al.^4^, who reported an association between genotypes at rs3813865 and CRC in Chinese populations.

In our investigation, we analyzed one of the most commonly studied SNPs in exon 4 of the *TP53* gene, rs1042522 (CCC changes to CGC), resulting in the Pro72Arg amino acid change. This nucleotide alteration disrupts the Proline-rich region of the p53 protein, impacting its normal function in cell cycle regulation and DNA repair, ultimately contributing to cancer development^12^. Consistent with these findings, our study revealed a higher frequency of homozygous mutant alleles (GG) in CRC cases, while heterozygous alleles (GC) were more frequent in controls (Table 1). Our results align with previous studies reporting an association between rs1042522 in *TP53* and CRC in subjects from Jammu and Kashmir^29^, Denmark^30^, China^10^, and Bangladesh^31^. Conversely, Asadi et al.^11^ and Polakova et al.^32^ reported no significant differences in allele prevalence at rs1042522 in the TP53 gene between patients and controls in Iranian Azari and Czech Republic populations, respectively.

The human *MDM2* gene, located on chromosome 12q13–14, possesses two promoters, a constitutive and a p53-responsive intronic promoter^33^. A common polymorphism (rs2279744, T309G) in the *MDM2* promoter has been shown to increase MDM2 mRNA and protein expression by altering Sp1-binding affinity, thereby inhibiting p53^34^. In our study, none of the genotypes at rs2279744 in MDM2 were found to be associated with colorectal cancer (CRC) (Table 1). Numerous studies have explored the association of rs2279744 in *MDM2* with CRC, yielding controversial and inconclusive results. Similar to our findings, Talseth et al.^35^ found no association of any genotype at rs2279744 with CRC in subjects from Australian and Polish populations. Alhopuro et al.^36^ also reported no association between genotypes at rs2279744 and CRC patients of Finnish origin. Conversely, Yueh et al.^37^, Atabey et al.^15^, and Liu et al.^38^ reported a significant association of rs2279744 in *MDM2* in Taiwanese, Turkish, and Chinese populations, respectively.

Results from studies examining individual SNPs are inconsistent, as each SNP alters the function of a single gene among many involved in carcinogenesis. Disease progression may result from the interaction of several proteins. Considering this, we performed SNP-SNP interaction analysis, exploring various possible SNP combinations from the four SNPs screened in our study to identify combinations most likely associated with CRC risk. Our results indicated that combinations of various SNPs at rs1799883 (*FABP2*) and rs3813865 (*CYP2E1*), rs1042522 (*TP53*), and rs2279744 (*MDM2*), as well as genotype combinations at rs3813865 (*CYP2E1*) and rs1042522 (*TP53*), were associated with a higher incidence of CRC. Our findings support those of Zhang et al.^39^, who reported that the combined effect of *TP53* Arg72Pro (rs1042522) and *MDM2* SNP309 (rs2279744) variant genotypes increased CRC risk in a Chinese population. Additionally, we identified two new SNP combinations associated with CRC development in our study, which should be further analyzed in other populations to confirm their association with CRC.

Analysis of the studied risk factors revealed that unmarried status, smokers, and subjects with exercise habits but no family history were at a higher risk of developing colorectal cancer (CRC). Numerous studies worldwide have reported risk factors associated with CRC across various ethnic groups. Bhattacharya et al.^3^ reported that individuals of Indian origin living in urban areas, those who were obese, smokers and those with a history of non-vegetarian dietary intake were more susceptible to CRC. Diet composition, especially one rich in N-nitroso compounds, has been documented as having a significant association with the development of gastrointestinal tract cancers^40^.

During the present study, it was also observed that males and patients older than 50 years were more susceptible to CRC than females and subjects younger than 50, although these differences did not reach statistical significance (Table 3). Our results align with Zubair et al.^24^, who reported that the age and sex of subjects were not associated with the development of CRC in individuals enrolled from Khyber Pakhtunkhwa in Pakistan. In contrast to our observations, Bhattacharya et al.^3^ reported a significantly increased risk of developing CRC with increasing age, with individuals older than 50 having a higher incidence of CRC in the Indian population^3^. Similar to our findings, Bhattacharya et al.^3^ also observed an increased risk of CRC in males compared to females, although the difference did not reach statistical significance.

The current study has several limitations that need addressing. First, we aimed to genotype as many patients and healthy individuals as possible. The larger the sample size, the more reliable the observations and results. However, we finally analyzed 100 CRC cases and 100 healthy controls. Secondly, the number of genes and SNPs related to CRC in our study was limited, whereas many other genes have been proven to be associated with CRC. There is a need to explore more genes and SNPs related to CRC to accurately assess susceptibility genes for CRC.

While our study provides valuable insights into the genetic factors associated with colorectal cancer (CRC) susceptibility in the Southern Punjab population, it is important to acknowledge certain limitations. The modest sample size of 100 CRC cases and 100 controls, although informative, suggests the potential for broader investigations to enhance statistical robustness and generalize findings more effectively. Our genetic focus on specific genes (*FABP2, CYP2E1, TP53*, and *MDM2*) and associated SNPs undoubtedly contributes to the understanding of CRC risk factors. However, there exists an opportunity to explore a wider array of genetic variations to uncover a more comprehensive picture. Geographically, our study concentrated on Southern Punjab, thereby offering insights specific to that region. While insightful, this regional focus may limit the broader applicability of findings. Future research should consider expanding to diverse regions in Pakistan to capture a more representative spectrum of CRC patterns. In terms of study design, the cross-sectional case–control approach suits genetic associations well. However, establishing causation necessitates longitudinal studies, an aspect for consideration in future research endeavors. Our study primarily delved into genetic factors, excluding detailed examinations of gene-environment interactions. Exploring these interactions could provide a more nuanced understanding of CRC risk factors. While we recognize these limitations, it is important to emphasize that our study forms a valuable foundation for understanding CRC susceptibility in the Southern Punjab population. The insights gained pave the way for future research that addresses these limitations and further refines our understanding of CRC in the Pakistani context.

## Conclusion

In conclusion, our study sheds light on the intricate interplay of genetic factors in colorectal cancer (CRC) susceptibility within the Southern Punjab population. The investigation identified significant associations between specific single nucleotide polymorphisms (SNPs) in FABP2, CYP2E1, TP53, and MDM2 genes and the risk of CRC development. Notably, the observed variations in genotypic frequencies underscore the ethnic diversity in CRC susceptibility, emphasizing the need for region-specific considerations in genetic studies. The uniqueness of our study lies in its focused examination of distinct SNPs within key genes associated with CRC, providing a foundation for understanding the genetic landscape of CRC in Southern Punjab. These findings contribute to the evolving field of personalized medicine, paving the way for tailored screening and preventive strategies based on an individual's genetic profile. Moving forward, prospective studies with expanded sample sizes, the inclusion of additional genetic markers, and the exploration of gene-environment interactions could unravel more intricate details of CRC etiology in the Pakistani context. Additionally, our results underscore the importance of incorporating genetic screening into comprehensive health planning, offering potential avenues for targeted interventions and improved CRC management in the region.

## Supplementary Information

Supplementary Figure 1.

## Data Availability

All data generated or analysed during this study are included in this submitted article.
