# Supplementary material for: Genetic polymorphisms in FABP2, CYP2E1, and TP53 genes are potentially associated with colorectal cancer susceptibility
Source: Sci Rep. 2024 Sep 3;14:20464. doi: 10.1038/s41598-024-70381-y (PMC11379967; doi:10.1038/s41598-024-70381-y)

**Supplementary Fig. 1.**

Agarose gel electrophoresis based genotyping results.

**Legend**

1. PCR-RFLP based genotyping at rs1799883 in *FABP2*. Lane L presents 100 bp DNA ladder. lane 1 -4 showing homozygous wild (GG) genotype (81 and 99 bp restriction products), lane 5-11 are presenting heterozygous (GA) genotype (81, 99 and 180 bp).

B. T-ARMS PCR based genotyping at rs3813865 in *CYP2E1*. Lane L presents 100 bp DNA ladder. Lane 1, 2 and 4 is showing heterozygous (GC) genotype (499, 303 and 236 bp) and lane 3, 5 and 6 is presenting homozygous mutant (CC) genotype (499 and 236 bp).

C. T-ARMS PCR based genotyping at rs1042522 in *TP53*. Lane L presents 100 bp DNA ladder. Lane 1, 3, 6, 8 and 9 presenting heterozygous (GC) genotype (493, 247 and 200 bp), lane 5, 8 and 9 presenting homozygous mutant (GG) genotype (493 and 200 bp).

D. T-ARMS PCR based genotyping at rs2279744 in *MDM2*. Lane L presents 100 bp DNA ladder. Lane 2 and 4 presenting heterozygous (TG) genotype (224, 158 and 122 bp). Lane 5 showing homozygous (GG) genotype (224 and 158 bp). While lane1represent homozygous wild (TT) genotype (224 and 122 bp).


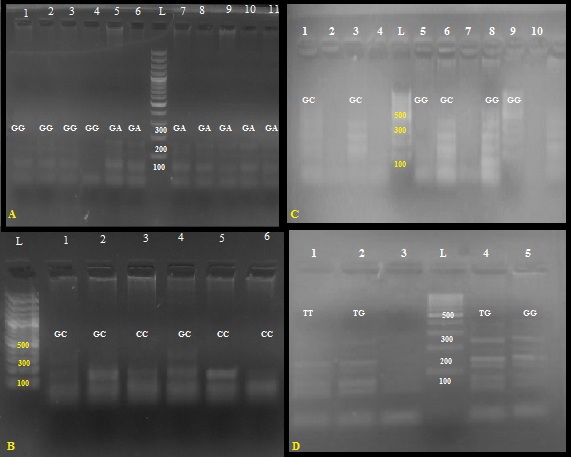

Supplement: Supplementary file 1 — Supplementary Figure 1. [file 41598_2024_70381_MOESM1_ESM.docx]
